# Recurrent and Subsequent Injuries in Professional and Elite Sport: a Systematic Review

**DOI:** 10.1186/s40798-020-00286-3

**Published:** 2020-12-03

**Authors:** Charlotte Leah Bitchell, Jo Varley-Campbell, Gemma Robinson, Victoria Stiles, Prabhat Mathema, Isabel Sarah Moore

**Affiliations:** 1 Cardiff School of Sport and Health Sciences, Cardiff Metropolitan University, Cardiff, UK; 2 University College London, London, UK; 3 University of Exeter, Exeter, UK; 4 Welsh Rugby Union Group, WRU National Centre of Excellence, Vale of Glamorgan, UK

**Keywords:** Systematic review, Professional sport, Recurrent, Subsequent, Injury

## Abstract

**Background:**

Injury surveillance in professional sport categorises injuries as either “new” or “recurrent”. In an attempt to make categorisation more specific, subsequent injury categorisation models have been developed, but it is not known how often these models are used. The aim was to assess how recurrent and subsequent injuries are reported within professional and elite sport.

**Methods:**

Online databases were searched using a search strategy. Studies needed to prospectively report injury rates within professional or elite sports that have published consensus statements for injury surveillance.

**Results:**

A total of 1322 titles and abstract were identified and screened. One hundred and ninety-nine studies were screened at full text resulting in 81 eligible studies. Thirty studies did not report recurrent injuries and were excluded from data extraction. Within the studies that reported recurrent injuries, 21 reported the number and percentage; 13 reported only the proportion within all injuries; three reported only the number; five reported the number, percentage and incidence; and two only reported the incidence. Seven studies used subsequent injury terminology, with three reporting subsequent injury following concussion, one using an amended subsequent injury model and three using specific subsequent injury categorisation models. The majority of subsequent injuries (ranging from 51 to 80%) were categorised as different and unrelated to the index injury. The proportion of recurrent injuries (exact same body area and nature related to index injury) ranged from 5 to 21%.

**Conclusions:**

Reporting recurrent or subsequent injuries remains inconsistent, and few studies have utilised subsequent injury models. There is limited understanding of subsequent injury risk, which may affect the development of injury prevention strategies.

**Trial Registration:**

CRD42019119264

## Key Points

- Reporting the recurrent or subsequent nature of an injury remains inconsistent within research.
- Thirty of the articles that achieved the criteria for data extraction did not report recurrent injuries.
- Only three studies utilised subsequent injury categorisation models to categorise subsequent injuries, with only two using the SIC 1.0 and one using the SIC 2.0.

## Introduction

Injury risk associated with participation in sport can vary depending on the type of sport and level of play [1–8]. One of the main priorities for any medical team in a professional or elite sporting environment is to reduce the number of days a player is unavailable throughout a season. Prospectively recording injuries can help inform the development of interventions aimed at injury prevention and rehabilitation [9, 10]. To improve the development of injury prevention programmes, a four-step framework identifying the injury problem and aetiology was initially developed by van Mechelen et al. [11]. Finch [12] proposed an updated framework in 2006, the Translating Research into Injury Prevention Practice (TRIPP), in order to continue building an evidence base for effective injury prevention. Both frameworks identify that using objective data from injury surveillance to establish the injury problem is the first stage of effective injury prevention [11, 12]. Consequently, consensus statements outlining definitions and procedures to follow when conducting injury surveillance have been created for athletics, aquatic sports, association football, cricket, horseracing, multi-sport events, rugby union and tennis to ensure consistency within sports [13–21].

The primary purpose of injury surveillance is to identify priority injury problems; however, a limitation with the current reporting of injury rates is the lack of distinction between new and recurrent injuries. Within traditional injury surveillance, all recorded injuries are new injuries unless they are the exact same injury as a previous one, in which case they are referred to as a recurrent injury [22]. Yet often no distinction is made in reported injury rates. The lack of clarity on whether an injury is new or recurrent makes developing prevention protocols challenging, particularly when considering the increased risk of injury following previous injury and increased severity of recurrent injuries [23–26]. Further challenges arise when players sustain multiple injuries, contributing multiple entries to the overall injury database. Whilst some studies identify that players have sustained multiple injuries during the injury surveillance period, they do not always provide any detail on the type or location of the injuries [27, 28]. Furthermore, there are often discrepancies between using multiple (several unrelated injuries), recurrent (more than one occurrence of the exact same injury) or subsequent (any injury occurring after the index injury, where the index injury represents the first recorded injury of an athlete within a surveillance period) terminology to describe the injury occurrence, despite the different definitions [8, 22, 29–32].

Although consensus statements have outlined a standard method of reporting recurrent injuries [13–21], the simple definition negates the potential for the reporting of subsequent injuries that do not present with the same diagnosis to be identified and analysed in research. Consequently, research has aimed to classify subsequent injuries as local, recurrent, re-injury or exacerbation and has provided more specific categories for a clinical and data-driven categorisation through subsequent injury categorisation models (SIC 1.0 and 2.0) [12, 22, 31–33]. The publication of standardised data collection procedures in consensus statements and specific subsequent injury categorisation models provide an opportunity for research to identify the recurrent or subsequent nature of injuries. However, it remains unknown whether injury surveillance research within professional and elite sport investigates recurrent or subsequent injuries, limiting the current understanding of the occurrence of these types of injuries.

This article systematically reviews the reporting of recurrent and subsequent injuries in prospective injury surveillance research within professional and elite sports that have a published, peer-reviewed consensus statement on the definitions and procedures for reporting injuries.

## Methods

The Preferred Reporting Items for Systematic Reviews and Meta-Analysis (PRISMA) statement was followed to ensure accurate reporting throughout [34]. The systematic review was prospectively registered on PROSPERO international prospective register for systematic reviews (CRD42019119264).

### Search

The online databases of SCOPUS, Embase (via Ovid) and Medline (via Ovid) were used to search for articles. The search strategy included articles published after 2005, the earliest publication date of the peer-reviewed consensus statement in the included sports [21], until 23 July 2020. The search was limited to English language articles, with the search on Embase and Medline limited to full text and human participants. Only sports with a published peer-reviewed consensus statement on the definitions and data collection procedures for reporting injuries were included in the search strategy. To ensure research reporting injuries in professional or elite sports was returned, the search strategy included injury and sports terms linked with Boolean operators “AND” and “OR”. The following terms were included in the search:

- Injury terms: injuries, injury, recurrent, subsequent
- Sport terms: football, rugby, athletics, swimming, cricket, diving, waterpolo, tennis, horseracing, professional, elite

The terms were entered into the search engine as follows: injury OR injuries AND recurrent OR subsequent AND football OR rugby OR athletics OR swimming OR cricket OR diving OR waterpolo OR tennis OR horseracing AND professional OR elite.

### Study Selection

All results returned by the online databases were exported to EndNote for the organisation of references and removal of duplicates before titles and abstracts were screened. Once duplicates had been removed, titles and abstracts of all remaining articles were initially screened for the inclusion of injury reporting in professional- or elite-level football (including Australian Football League, soccer and Gaelic football), rugby (including rugby union, rugby league and rugby sevens), athletics, aquatic sports (including swimming, waterpolo, diving and biathlon), cricket, tennis and horseracing (injuries to the jockey only). Conference abstracts, commentaries and systematic and literature reviews were excluded, and only full-text articles were eligible. The selection of articles identified for full-text screening were then screened to identify whether they included the following: (1) prospectively collected data, which provides a more accurate method of data collection when recording the exposure and injury details within sport [35]; (2) data collection procedures and definitions following a consensus statement; (3) injury records maintained by one designated medical team member for consistency within injury recording; and (4) data on professional- or elite-level sport. Articles reporting injuries in amateur-level sport were excluded to enable a consistent comparison across the same level of sport. When the original full-text article could not be located, authors were contacted directly. The reference list of included articles was also searched to identify further appropriate studies. An initial sample of 10% of titles and abstracts (*n* = 170) was screened by the primary (CLB) and second author (JV-C). As there was an almost perfect agreement between authors (Cohen’s *K* 0.97), the primary author screened all remaining titles and abstracts. All full-text articles outlined for each stage of review following the screening of titles and abstracts were reviewed by both authors independently (CLB and JV-C). If there were any discrepancies between the authors on the inclusion of an article, these were discussed, and if no agreement was made, a third author (ISM) was used as an adjudicator. There was an almost perfect agreement on the full-text articles screened (Cohen’s *K* 0.83). Discussion on inclusion resolved any disagreement between authors.

### Data Extraction

Data were extracted from the eligible full-text articles and recorded in an Excel spreadsheet. The extracted data contained information on (1) how the data were collected (i.e. prospective cohort); (2) injury definitions and procedures used, including how new and recurrent injuries were defined; (3) length of data collection period; (4) number of teams or players and level of play; (5) sex of the participants; (6) number of injuries sustained overall, and where relevant the match and training injury incidence; and (7) number of recurrent or subsequent injuries, including the subsequent injury category, the recurrent injury rate and the type or severity of recurrent injuries. As the search criteria included only professional and elite sports with consensus statements, all articles included followed the injury definitions and data collection procedures of the respective sports consensus statements. Definitions of injury and recurrent injury in each of the sports consensus statements are shown in Table 1. Study quality was assessed using an amended Downs and Black [36] checklist. The checklist was amended to exclude questions associated with confounders and intervention studies (questions 5, 8, 13 and 25) due to the prospective nature of data collection within the studies.

**Table 1:** The definitions of a new injury and recurrent injury from consensus statements

References	Sport	Injury definition	Recurrent injury definition
**Orchard et al., 2005** [21]	Cricket	Any injury or other medical condition that either (a) prevents a player from being fully available for selection for a major match or (b) during a major match, causes a player to be unable to bat, bowl or keep wicket when required by either the rules or the team’s captain.	A recurrent injury is one to the same side and body part and of the same injury type as an injury that previously qualified as a significant injury earlier in the same season, but which had recovered.
**Fuller et al., 2006** [14]	Football	Any physical complaint sustained by a player that results from a football match or football training, irrespective of the need for medical attention or time-loss from football activities. An injury that results in a player receiving medical attention is referred to as a “medical-attention” injury and an injury that results in a player being unable to take a full part in future football training or match play as a “time-loss” injury.	An injury of the same type and at the same site as an index injury and which occurs after a player’s return to full participation from the index injury. A recurrent injury occurring within 2 months of a player’s return to full participation is referred to as an “early recurrence”, one occurring 2 to 12 months after a player’s return to full participation as a “late recurrence” and one occurring more than 12 months after a player’s return to full participation as a “delayed recurrence”.
**Fuller et al., 2007** [13]	Rugby	Any physical complaint, which was caused by a transfer of energy that exceeded the body’s ability to maintain its structural and/or functional integrity, that was sustained by a player during a rugby match or rugby training, irrespective of the need for medical attention or time-loss from rugby activities. An injury that results in a player receiving medical attention is referred to as a “medical-attention” injury and an injury that results in a player being unable to take a full part in future rugby training or match play as a “time-loss” injury.	An injury of the same type and at the same site as an index injury and which occurs after a player’s return to full participation from the index injury. A recurrent injury occurring within 2 months of a player’s return to full participation is referred to as an “early recurrence”, one occurring 2 to 12 months after a player’s return to full participation as a “late recurrence” and one occurring more than 12 months after a player’s return to full participation as a “delayed recurrence”.
**Junge et al., 2008** [16]	Multi-sport (IOC)	Any musculoskeletal complaint newly incurred due to competition and/or training during the tournament that received medical attention regardless of the consequences with respect to absence from competition or training.	An injury of the same location and type, which occurs after an athlete’s return to full participation from the previous injury.
**Pluim et al., 2009** [19]	Tennis	Any physical or psychological complaint or manifestation sustained by a player that results from a tennis match or tennis training, irrespective of the need for medical attention or time-loss from tennis activities.	A medical condition of the same type and at the same site linked to an index medical condition and which occurs after a player’s return to full participation from the index medical condition.
**Turner et al., 2012** [20]	Horseracing	Any physical complaint sustained by a person that results from competitive riding, training or other recognised activity that brings a person into contact, or in close vicinity and with the potential for contact, with one or more thoroughbred racehorses, irrespective of the need for medical attention or time-loss from horseracing activities.	An injury of the same type and at the same site as an index injury, and the one that occurs after a person’s return to full participation in equine-related activities following the index injury.
**Timpka et al., 2014** [18]	Athletics	A physical complaint or observable damage to body tissue produced by the transfer of energy experienced or sustained by an athlete during participation in athletics training or competition, regardless of whether it received medical attention or its consequences with respect to impairments in connection with competition or training. A time-loss injury or illness is one that leads to the athlete being unable to take full part in athletics training and/or competition the day after the incident occurred.	An incident of the same type and at the same site linked to an index incident and which occurs after an athlete’s return to full function and participation from the index recordable incident.
**Mountjoy et al., 2015** [17]	Aquatic	A physical complaint or observable damage to body tissue produced by the transfer of energy experienced or sustained by an athlete during participation in training or competition in an aquatic discipline, regardless of whether it received medical attention or its consequences with respect to impairments in competition or training. A time-loss injury or illness leads to the athlete being unable to take full part in FINA activities.	Injury to same location and of the same type as the index injury, where the index injury has completely healed.
**Orchard et al., 2016** [15]	Cricket	A general time-loss injury is any injury (or illness) that results in a player being considered unavailable for match play, irrespective of whether a match or training was actually scheduled.	A recurrent injury is one of the same type which reoccurs in the same season (surveillance year) after it has been defined as recovered.

### Reporting of Results

Extracted data were summarised for each article. The methods of reporting injuries, the number and incidence of recurrent injuries and the proportions and categories for subsequent injuries were collated in order to provide a narrative overview of results. When analysing and reporting injuries in the current review, combining new and recurrent injuries was defined as the total injury rate.

## Results

**Fig. 1:**
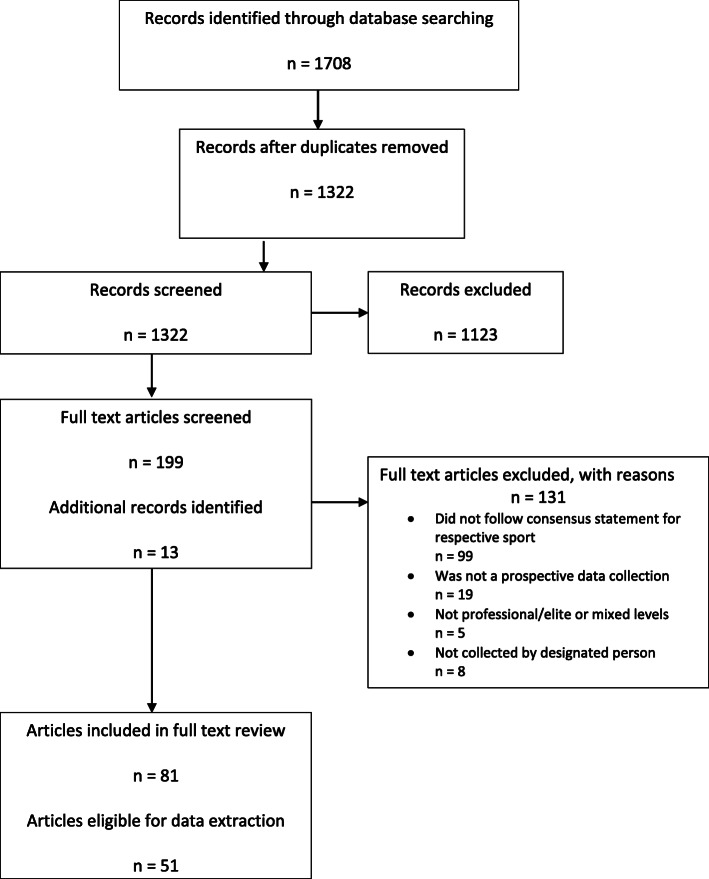
PRISMA flow diagram

The online database search returned 1708 articles (Fig. 1). Once duplicates were removed, 1322 titles and abstracts were screened, and 199 articles met the inclusion criteria outlined for this stage. The 199 articles were eligible for full-text screening, and an additional 13 articles were identified in references. Of the 212 full-text articles screened, 81 articles met the inclusion criteria. However, 30 (37%) of these articles did not report the recurrence of injuries within the study, therefore resulting in 51 articles (one article containing two studies) being eligible for data extraction.

The number of studies within each sport varied, with 31 studies in football/soccer [4, 25, 30, 37–64], 13 in rugby (two rugby sevens, eleven rugby union) [2, 27, 65–75], five in athletics [76–80], one in Olympic multi-event [81] and one in cricket [70] (Table 2). There were no studies from aquatic sports, horseracing or tennis. Although there were a range of sports included, only one study reported injury rates specifically in female sport [69]. The duration of data collection ranged from 3 days [79] to 16 seasons [59, 64]. In the studies where data collection was conducted across multiple seasons, studies often did not clarify how many athletes were involved each season, instead opting to report the overall number of athletes involved across all seasons [25, 41, 49, 51, 56, 64]. There was a wide range in the number of participants included in the data collection, from 36 athletes [39] to 9672 athletes [81], whilst nine studies only reported the number of teams without providing the number of individual athletes that participated [37, 42, 44, 47, 51, 70]. Only ten studies reported the number of athletes sustaining the total number of injuries [30, 41, 47, 50, 56, 65, 70, 72, 75], and two studies reported the proportion of players who sustained more than one injury [27, 30]. The majority of studies (*n* = 37) used a time-loss definition for injury, five used a medical attention definition, five used a medical attention and time-loss definition and four used an all-encompassing definition. The number of injuries sustained within the studies ranged from 15 injuries across 3 years within 552 athletes [69] to 22,942 injuries across one to 16 seasons within 116 teams [59].

**Table 2:** Description of the quality of the article, data collection period, sex of participants, sport, number of teams or athletes/players, injury definition used within the study and injury data reported

References	Data quality	Data collection	Sex	Sport	Number of teams/players	Injury definition	Number of injuries (incidence)	Number of recurrent or subsequent injuries (proportion)	Recurrent injury incidence	Subsequent injury category
**van Beijsterveldt et al.** [60]	16/23	Continuous prospective 6 years	Male	Football	14 clubs	Time-loss	2365 (4.8/1000 h)	20% (58% of muscle injuries were recurrent)	–	–
**Bengtsson et al.** [64]	11/23	Prospective cohort 16 seasons	Male	Football	64 teams 4088 players	Time-loss	16,087 (25.0/1000 match hours)	219	46.9 injuries/1000 match hours	–
**Bjørneboe et al.** [37]	13/23	Continuous prospective 6 years	Male	Football	14 clubs	Time-loss	2365 (4.8/1000 h)	20%	–	–
**Dvorak et al.** [38]	12/23	Prospective Up to 1 month	Male	Football	32 teams 736 players	All encompassing	125 match (time-loss = 40.1/1000 match hours) 104 training (time-loss = 4.4/1000 training hours)	12 (11.5%)	–	–
**Eirale et al.** [39]	14/23	Prospective 17 months	Male	Football	36 players	Time-loss	78 (78.0/1000 match hours)	19 (24.4%)	1.9/1000 h	–
**Eirale et al.** [40]	14/23	Prospective cohort 3 seasons	Male	Football	609 players	Time-loss	826 (4.97/1000 h)	97	–	–
**Ekstrand et al.** [44]	13/23	Prospective cohort 7 seasons	Male	Football	14 teams	Time-loss	4483	12%	–	–
**Ekstrand et al.** [43]	11/23	Prospective cohort 4 seasons	Male	Football	23 teams 816 players	Time-loss	516	16%	–	–
**Ekstrand et al.** [25]	11/23	Prospective, throughout season 1–9 seasons	Male	Football	51 teams 2299 players	Time-loss	2908	16%	–	–
**Ekstrand et al.** [46]	9/23	Prospective, throughout season 8 seasons	Male	Football	54 teams 2379 players	Time-loss	51	29%	–	–
**Ekstrand et al.** [41]	14/23	Prospective 1–12 seasons	Male	Football	3487 players	Time-loss	67 fractures (0.037/1000 match hours)	7 refractures (25%)	–	–
**Ekstrand et al.** [45]	12/23	Prospective cohort 1 season	Male	Football	31 teams 1032 players	Time-loss	393	49 (12%)	–	–
**Ekstrand et al.** [47]	11/23	Prospective, throughout seasons 8 seasons	Male	Football	46 teams	Time-loss	1488	16%	–	–
**Ekstrand et al.** [42]	11/23	Long-term prospective observational 13 seasons	Male	Football	36 clubs	Time-loss	1614 (1.2/1000 h)	216 (13%)	–	–
**Ekstrand et al.** [59]	8/23	Prospective 1–16 seasons	Male	Football	116 teams	Time-loss	22,942	3016 (1.3–48%)	–	–
**Ergün et al.** [48]	10/23	Prospective 3 years	Male	Football	52 players	Medical attention and time-loss	44 injuries—29 time-loss (30.4 match time-loss injuries/1000 h)	11 (25%)	–	–
**Gajhede-Knudsen et al.** [49]	10/23	Prospective 1–11 seasons	Male	Football	27 teams 1743 players	Time-loss	8029	12% (27% of Achilles injuries were recurrent)	–	–
**Gomez-Piqueras et al.** [50]	13/23	Prospective 2 seasons	Male	Football	1 team 71 players	All encompassing	165	12 (22%)	–	–
**Hägglund et al.** [62]	11/23	Prospective cohort 9 seasons	Male	Football	51 teams 2299 players	Time-loss	139	1 in 5	–	–
**Hagglund et al.** [61]	14/23	Prospective 9 seasons	Male	Football	26 clubs 1401 players	Time-loss	6140	564 (27%)	–	–
**Hägglund et al.** [4]	11/23	Prospective 1–14 seasons	Male	Football	43 top-level 19 elite	Time-loss	11,581 top-level (7.2/1000 h), 3836 elite (7.4/1000 h)	1615 (17%) top-level, 794 (25%) elite	1.00/1000 h top-level, 1.52/1000 h elite	–
**Gajhede-Knudsen et al.** [49]	10/23	Prospective 1–11 seasons	Male	Football	27 teams 1743 players	Time-loss	8029	12% (27% of Achilles injuries were recurrent)	–	–
**Larruskain et al.** [63]	13/23	Prospective 6 seasons	Male	Football	107 players	Time-loss	160 injuries (1.64/1000 h—7.49/1000 match hours, 0.71/1000 training injuries)	64 (24 (15%) less than 2 months, 40 (25%) within same season)	0.25/1000 h less than 2 months, 0.41/1000 h within same season	–
**Larruskain et al.** [53]	14/23	Prospective 5 seasons	Mixed	Football	85 players	Time-loss	483 (8.31/1000 h men, 6.3/1000 h women)	135 (31% for men, 23% for women)	2.55 recurrence/1000 h for men, 1.42/1000 h for women	–
**Hallen and Ekstrand** [51]	12/23	Prospective 1–12 seasons	Male	Football	89 teams	Time-loss	17,371, 5603 muscle injuries	15%	–	–
**Jones et al.** [52]	10/23	Prospective cohort 1 season	Male	Football	10 teams 243 players	Time-loss	473 (9.11/1000 h)	27.3% hamstring, 20% groin, 10% quads	–	–
**Lee et al.** [54]	12/23	Prospective 1 season	Male	Football	7 teams 152 players	Time-loss	296 (7.4/1000 h—61.1 for match, 3.4 for training)	52 (21%) 29% early recurrence, 39% late recurrences, 32% delayed recurrence	–	–
**Lundblad et al.** [55]	10/23	Prospective cohort 11 seasons	Male	Football	27 teams 1743 players	Time-loss	8029—346 MCL injuries (0.33/1000 h—1.31 match, 0.14 training)	11%	–	–
**Nordström et al.** [56]	11/23	Prospective observational 1–11 seasons	Male	Football	46 teams 1665 players	Time-loss	8695—71 concussions	756 subsequent to concussion	–	–
**Noya Salces et al.** [57]	11/23	Prospective, throughout season 1 season	Male	Football	16 clubs 427 players	All encompassing	1293—524 match (43.5/1000 h), 769 training (3.6/1000 h)		4.7 in match, 0.4 in training per 1000 h	–
**Petersen et al.** [58]	11/23	Prospective, throughout season 12 months	Male	Football	16 teams 374 players	All encompassing	46—28 in match (1.82/1000 h), 14 in training (0.12/1000 h)	8 (25%)	–	–
**Stubbe et al.** [30]	13/23	Prospective 39 weeks	Male	Football	8 teams 217 players	Time-loss	286 (6.2/1000 h)	8% 76 players sustained multiple—40 injured twice, 16 injured 3 times, 11 injured 4 times, 9 injured 5 times or more	0.5 recurrence/1000 h	–
**Cross et al.** [65]	11/23	Prospective cohort 2 seasons	Male	Rugby	810 players	Time-loss	181 (8.9/1000 h)	–	116.1—144.6/1000 h	–
**Fuller et al.** [66]	13/23	Prospective, throughout tournament 7 weeks	Male	Rugby	626 players	Time-loss	161 match, 60 training (83.9/1000 h)	9 match, 16 training	–	–
**Fuller et al.** [67]	14/23	Prospective, throughout tournament 7 weeks	Male	Rugby	615 players	Time-loss	171 match, 35 training (89.1 match, 2.2 training injuries/1000 h)	24 (14%) match, 17% training	–	–
**Fuller et al.** [2]	14/23	Prospective, throughout tournament 7 weeks	Male	Rugby	639 players	Time-loss	173 match, 20 training (90.1 match, 1.0 training injuries/1000 h)	20 (11.6%) match, 3 (15%) training	–	–
**Kenneally-Dobrowski et al.** [74]	11/23	Prospective 5 years	Male	Rugby	74 players	Time-loss	30 hamstring injuries—63% training, 37% match	7%	–	–
**Kemp et al.** [68]	10/23	Prospective, weekly throughout season 3 seasons	Male	Rugby	13 clubs 757 players	Time-loss	155 match, 14 training. 96 match concussions, 5 training	10%	–	–
**Ma et al.** [69]	14/23	Prospective cohort 3 years	Female	Rugby	552 players	Medical attention and time-loss	15 (32.6/1000 h)	24%	–	–
**Moore et al.** [27]	13/23	Prospective, throughout tournament 3 years	Male	Rugby	1 team 78 players	Time-loss	144 match (180.0/1000 h), 41 training (4.7/1000 h)	19%	–	–
**Moore et al.** [70]	10/23	Prospective 3 seasons	Male	Rugby	1 team	Time-loss	648	29% were subsequent	–	59% SIC 10 21% SIC 2, 3 or 4
**Pearce et al.** [71]	9/23	Prospective 4 seasons	Male	Rugby	899 players	Time-loss	147 (3.3/1000 match hours, 0.09/1000 training hours)	15–20%	–	–
**Rafferty et al.** [72]	10/23	Prospective, throughout season 4 years	Male	Rugby	4 teams 429 players	Time-loss	2441 injuries—1602 match (94.5—177.0/1000 h), 514 training	18% greater risk of injury after concussion	–	–
**Toohey et al.** [75]	12/23	Prospective cohort 2 seasons	Mixed	Rugby	90 players	Medical attention	365 43.2/1000 h	95.2% players sustained at least 1 subsequent injury	–	80.7% SIC VIII, 10.3% SIC VII, 6.1% SIC VI
**Williams et al.** [73]	10/23	Prospective cohort 8 seasons	Male	Rugby	1556 players	Time-loss	9597 time-loss—6903 match, 2617 training	8180 (85%) subsequent injuries—6063 in match, 2087 in training	–	70% were “new” 14% were “local” 16% were “recurrent”
**Junge et al.** [81]	10/23	Prospective, throughout championships 16 days	Mixed	Multi-events	92 teams 9672 athletes	Medical attention	1055 (96.1 per 1000 registered athletes)	47 (5.5%)	–	–
**Alonso et al.** [76]	11/23	Prospective 8 days	Mixed	Athletics	49 teams 1980 athletes	Medical attention	105 time-loss injuries (53.0 time-loss per 1000 athletes)	15 (8%)	–	–
**Alonso et al.** [77]	13/23	Prospective 9 days	Mixed	Athletics	47 teams 1486 athletes	Medical attention	236 (135.4/1000 registered athletes)	10.6%	–	–
**Alonso et al.** [78]	15/23	Prospective 9 days	Mixed	Athletics	61 countries 1512 athletes	Medical attention	249 (134.5/1000 registered athletes)	23 (9.3%)	–	–
**Edouard et al.** [79]	14/23	Prospective, throughout championship 3 days	Mixed	Athletics	440 athletes	Medical attention and time-loss	30 injuries—8 time-loss (47.5/1000 registered athletes)	1	–	–
**Edouard et al.** [80]	15/23	Prospective, during championship 5 days	Mixed	Athletics	1244 athletes	Medical attention and time-loss	132 (98.4/1000 registered athlete, 46.2 time-loss/1000 registered athletes)	8 (6.1%)	–	–
**Moore et al.** [70]	10/23	Prospective 3 years	Male	Cricket	1 team	Medical attention and time-loss	286—96 time-loss	90% were subsequent	–	51% SIC 10 8% SIC 7 or 8 5% SIC 2, 3, 4 or 6

### Risk of Bias Assessment

Scores ranged from 8 to 16, with a maximum possible score of 23. Studies with a lower quality score typically failed to report the following: (1) the participant characteristics (*n* = 34), (2) whether there was any attempt at blinding the participants (*n* = 50) and (3) whether there was any loss to follow-up (*n* = 46).

### Recurrent Injuries

All recurrent injury definitions were compliant with the consensus statement for the respective sport and are shown in Table 1. Of the 51 studies (one of the 51 articles contained two studies [70]), 44 reported recurrent injuries as either a number, percentage, incidence or a combination of these. Almost half of the articles, 21 of the 44, reported both the number and the percentage of recurrent injuries [2, 25, 38, 41–43, 45, 47, 48, 50, 54, 55, 58, 59, 61, 64, 67, 76, 78, 80, 81]; 13 only reported the percentage [27, 37, 44, 46, 49, 51, 52, 62, 68, 69, 71, 74, 77]; three only reported the number [40, 66, 79]; five reported the number, percentage and incidence [4, 30, 39, 53, 63]; and two only reported the incidence [57, 60]. The number of recurrent injuries ranged from one [79] to 3016 [59], the proportion ranged from 5.5 [81] to 48% [59] and the incidence ranged from 0.5 to 2.55 recurrent injuries per 1000 h [37, 53]. Only 16 of the 44 studies reporting recurrent injuries provided further detail regarding the severity of injury, with one study reporting the number and percentage of recurrent injuries that were time-loss injuries [76], one study reporting the proportion of total days lost due to recurrent injuries [27], two studies reporting the time lost for recurrent injuries [77, 78] and 12 comparing the severity of new and recurrent injuries [25, 30, 43, 44, 46, 48, 50, 54, 55, 68, 71]. Of the 12 studies comparing the severity of new and recurrent injuries, seven studies reported recurrent injuries to be more severe than new injuries [25, 30, 44, 46, 68, 71], four studies reported no differences in severity [43, 50, 54, 55] and one study reported recurrent injuries to be less severe than new injuries [48].

### Subsequent Injuries

Although subsequent injury categorisation models have been published since 2011 [32], only seven of the 50 studies used the subsequent injury terminology. Three of the seven studies analysed injuries sustained subsequent to concussion, where two studies reported the risk of injury following a concussion using a hazard ratio (1.38 [72], and 1.45 to 4.07 between 0 and 12 months [56]) as well as the median days to the next injury and the total number of injuries following a concussion (36 median days to next injury [72] and 153 injuries subsequent to concussion [56]). The third study reported the incidence of subsequent injury for players who returned from concussion in 14 days or less or more than 14 days (116.1 and 144.6/1000 h, respectively [65]), along with the median time to subsequent injury following concussion (53 days to subsequent injury [65]). One of the seven studies modified the subsequent injury classification from Hamilton and colleagues [32] to provide three options for subsequent injury, namely “new”, “local” and “recurrent” [73]. Within this study, 85% of injuries were classed as subsequent injuries, with 70% of the subsequent injuries reported as new injuries, 14% as local and 16% as recurrent. Two of the seven studies utilised the SIC 1.0 model [22] to report the percentage of subsequent injuries [70], where 89–91% of injuries were categorised as subsequent, rather than an initial injury [70]. The studies were also in agreement that the majority of subsequent injuries were categorised as SIC 10 (injury to different body part, unrelated to an index injury) with proportions ranging from 51 to 59% [70]. Although injuries were also classified in other categories, the proportion of these injuries was comparatively lower. Within the sport of rugby, 21% of subsequent injuries were categorised as SIC 2, 3 or 4 (same body site and nature, related to index injury) and none were categorised as SIC 5 (same body site and nature, unrelated to an index injury). Within cricket, 15% of subsequent injuries were coded as SIC 7 or 8 (same body site but different nature, related or unrelated to index injury); 14% were SIC 2, 3, 4 or 6 (same body site and nature, related or unrelated to index injury); and none were categorised as SIC 5 (same body site and nature, unrelated to an index injury) [70]. The seventh study utilised an updated version of the SIC 1.0 model outlined by Finch and Cook [22] which included a data-driven category for classifying subsequent injuries retrospectively [31]. Within this study, 81% of subsequent injuries were SIC 2.0 VIII (injury to different site and of different nature), 10% were SIC 2.0 VII (different site, same nature), 6% were SIC 2.0 VI (same site, different nature) and less than 3% were categories II–V (re-injury after recovery, same site, nature, side and structure; acute exacerbation before recovery, same site, nature, side and structure; injury of same site, nature, side; injury of same site and nature) [75].

## Discussion

This systematic review aimed to identify how recurrent or subsequent injuries have been reported across professional and elite sports. Consensus statements have been published to provide professional and elite sport with guidelines for data collection procedures and injury definitions, allowing more consistent comparisons across sport [13–21]. Furthermore, subsequent injury categorisation models have been published in order to provide researchers and clinicians with a more accurate definition of subsequent injury [22, 31–33]. However, the current review identified that there remains disparity both within and between sports on the methods utilised when reporting recurrent or subsequent injuries.

### Reporting Injuries

An important finding identified in the current review was that studies often analysed and presented injury data as pooled values across a range of seasons or years and within a large range of athletes. A similar finding was identified by Fortington and colleagues [28], where the systematic review highlighted that the majority of the studies analysed pooled injury data across teams and seasons. Furthermore, studies with a lower quality score according to the Downs and Black [36] assessment in the current review were often studies that pooled data across a number of seasons without specifying the number of new participants each season. The consistent reporting of pooled injury data across multiple participants or seasons fails to consider differences between individual athletes across seasons and could impact the analysis and interpretation of injury data. This is emphasised by the lower quality score identified in the Downs and Black [36] assessment and suggests that injury rates within these types of studies should be interpreted with caution due to the lack of specificity across the numerous seasons. Specifically, injury rates from studies in football [25, 37, 39, 41–44, 46–50, 53, 55, 56, 59, 61–64], rugby [27, 65, 68–75] and cricket [70] suffer from this problem.

In the current review, there were also variations in the data collection period used between studies, where some athletes were followed for less than a week in athletics and others followed for up to 16 seasons in football [59, 79, 80]. Within the studies where data collection extended across seasons, there was often a lack of information on the number of participants involved in each season [4, 25, 41, 49, 51, 55, 56, 59]. Furthermore, although the majority of the studies prospectively collected data over more than one playing season (67%), five studies collected injury data in a championship environment that often lasted less than 10 days [76–80]. As subsequent injuries have been reported to occur as late as 114 days following initial injury in previous research [65], the lack of a follow-up period within championship environments has the potential to under-report the risk of sustaining a subsequent injury from continued participation. In addition, reporting injuries sustained within a season alone fails to consider the influence of pre- or post-season on the reporting of subsequent injuries [28]. For example, if a study was to conduct injury surveillance with a team across a season and a player sustained an injury in the pre-season, any injury during the season could be considered a subsequent injury. However, if injury surveillance is conducted within the in-season period alone, any subsequent injuries sustained during the season would be inaccurately represented. Both the short timeframe of data collection and potential for out of season injuries to be missed suggest that standard recommendations associated with reporting recurrent or subsequent injuries should be established. Ensuring that the data collection period enables the accurate capturing of subsequent injuries would allow more consistent reporting between sports and improve the understanding of recurrent and subsequent injury risk.

### Definition of Recurrent Injuries

The definition of recurrent injuries outlined in consensus statements is similar between sports, therefore allowing a more consistent comparison between the rate of recurrent injuries reported. However, there remain inconsistencies in the way recurrent injuries are reported. One of the main findings in this systematic review was that 30 of the articles were excluded from data extraction as they failed to report recurrent injuries at all, even when following the definitions and data collection procedures outlined by a consensus statement. Within the studies that did report injury recurrence, a number of studies only identified the proportion within the total number of injuries [27, 37, 44, 46, 49, 51, 52, 60, 62, 68, 69, 74, 77], providing insufficient detail about the nature or consequence of the recurrent injuries. Additionally, only ten studies provided detail of the body area associated with recurrent or subsequent injuries, meaning appropriate (re)injury prevention strategies cannot be recommended. The lack of specificity with recurrent injury diagnosis becomes more of an issue when considering the contribution of previous injury to injury risk, with research identifying that previous injuries, often as long as 3 years prior to a new injury, significantly increase current injury risk [23, 26, 82]. Furthermore, within studies reporting the recurrence of injuries, diagnosis may rely on the clinician’s (and athlete’s) ability to recall the injury history of the athlete. Although it is not discussed in any of the prospective injury surveillance studies, recurrent injury categorisation is technically *retrospective* and possibly prone to recall bias and lack of awareness of injury history. This latter aspect can be potentially mitigated against in sports settings where the clinician has worked with the athletes for a sustained period of time. However, the issue surrounding the *retrospective* nature cannot be eliminated unless clinicians actively look at the athlete’s injury records in the injury surveillance system, or utilise subsequent injury models developed for the application of categorising subsequent injuries in sport [22, 31–33].

### Subsequent Injuries

In an attempt to reduce the recall bias and potential lack of awareness associated with diagnosing recurrent injuries, a data-driven categorisation that can be applied post hoc to the injury data that is not reliant on specific clinical knowledge during the data analysis has been suggested [31]. Following the subsequent injury model outlined by Hamilton and colleagues [32], Finch and Cook [22] developed a more specific categorisation of subsequent injuries that facilitates the identification of potential individual injury dependencies (SIC 1.0). In an attempt to improve the application of subsequent injury diagnoses, Toohey and colleagues [31] adapted the SIC 1.0 categorisation model to create the SIC 2.0 categorisation model, encompassing a clinically driven approach and data-driven approach that can be retrospectively applied to injury data. However, the limited uptake of the subsequent injury categorisation models in the current review means there is still a lack of specificity when reporting recurrent or subsequent injuries. Whilst the first categorisation of subsequent injuries was published in 2011 [32], 90% (*n* = 36) of the studies published after 2011 in the current study failed to categorise subsequent injuries. Although the SIC 1.0 and 2.0 were utilised in three studies in the current review, the study by Williams and colleagues [73] used a modified version of a previous classification system [73], which aimed to simplify the subsequent injury diagnosis based on the type and location of injury alone. The majority of the subsequent injuries sustained in the study by Williams and colleagues [73] were categorised as different injuries to the index injury, and the remaining injuries were of the same type or location. Although comparisons can still be made between studies utilising either simplified subsequent injury models or the SIC 1.0 and 2.0, the specificity of the SIC models allows researchers and clinicians to gain a better understanding of subsequent injuries and encourages the development of more specific prevention protocols [31, 70].

The accurate diagnosis of subsequent injuries can have a significant clinical impact, especially when considering the influence of previous injuries on sustaining new injuries [26, 83, 84]. Three of the seven studies using the subsequent injury terminology specifically focused on the next injury after a concussion [56, 65, 72], demonstrating that concussive injuries were associated with an increased risk of sustaining subsequent injuries. In addition to an increased risk, the studies by Cross and colleagues [65] and Rafferty and colleagues [72] demonstrated that there was a reduced number of days before a subsequent injury following concussion when compared with non-concussive injuries. Although this provides clinicians with important information regarding recovery from concussive injuries, the lack of detail in research associated with subsequent injuries following other types of injuries limits the potential for understanding the relationship with different types of injuries. As previous research has shown that a history of previous injury is positively associated with sustaining future injury [23, 26], exploring relationships between subsequent injuries sustained following different types of injuries could inform clinicians on the potential patterns between new and subsequent injuries, further aiding the development of injury prevention protocols.

## Limitations

A limitation in the current study was that the search strategy was limited to English language, meaning that articles reporting recurrent or subsequent injuries in other languages would not have been included. A further limitation was the restriction of studies within professional- or elite-level sports. The restriction to professional or elite sport resulted in studies reporting injuries within amateur and collegiate athletes being excluded from the review, even if recurrent injuries had been reported. However, ensuring only one level of sport was included in the review provides consistency both with the accuracy of injury diagnosis by professional medical provision and data collection procedures following a consensus statement for accurate comparisons between studies. Further research could incorporate all levels of sports, making comparisons between injury data to demonstrate whether discrepancies exist between playing levels. In addition, the results from some of the studies should be interpreted with caution due to the low score in the Downs and Black [36] assessment. Whilst the prospective nature of injury surveillance research makes aspects such as blinding participants and medical personnel challenging, the pooling of injury data across multiple seasons could influence the analysis and interpretation of injury rates. For example, pooling injury data could mask the potential difference in injury rates between individual athletes that take part in one season, but not another. This could consequently influence the overall injury rate reported within each season.

## Conclusion

Reporting the recurrent or subsequent nature of an injury remains inconsistent within research, even with the publication of consensus statements and the subsequent injury categorisation models. Furthermore, only a few studies have utilised subsequent injury categorisation models to accurately categorise subsequent injuries, meaning that risk of subsequent injury following an initial injury remains unclear. The lack of recurrent and subsequent injury reporting shows research is not providing an adequate understanding of the injury risk, meaning that injury prevention protocols to mitigate against recurrent and subsequent injuries may be insufficient. As injury prevention relies on accurate injury surveillance data, utilising the SIC model in future research will allow clinicians and researchers to distinguish between new and recurrent or subsequent injuries and improve our understanding of the role of inter-injury relationships in tertiary prevention strategies.

## Data Availability

Not applicable.

## Abbreviations

TRIPP: Translating Research into Injury Prevention Practice
SIC: Subsequent injury categorisation

## References

[1] Bahr R, Holme I (2003). Risk factors for sports injuries—a methodological approach. Br J Sports Med.

[2] Fuller CW, Taylor A, Kemp SPTT, Raftery M (2017). Rugby World Cup 2015: World Rugby injury surveillance study. Br J Sports Med.

[3] Giroto N, Hespanhol Junior LC, Gomes MRC, Lopes AD (2017). Incidence and risk factors of injuries in Brazilian elite handball players: a prospective cohort study. Scand J Med Sci Sports.

[4] Hagglund M, Walden M, Ekstrand J (2016). Injury recurrence is lower at the highest professional football level than at national and amateur levels: does sports medicine and sports physiotherapy deliver?. Br J Sports Med.

[5] Lathlean TJH, Gastin PB, Newstead SV, Finch CF (2018). The incidence, prevalence, severity, mechanism and body region of injury in elite junior Australian football players: a prospective cohort study over one season. J Sci Med Sport.

[6] Orchard JW, James T, Portus MR (2006). Injuries to elite male cricketers in Australia over a 10-year period. J Sci Med Sport.

[7] Roe M, Murphy JC, Gissane C, Blake C (2018). Time to get our four priorities right: an 8-year prospective investigation of 1326 player-seasons to identify the frequency, nature, and burden of time-loss injuries in elite Gaelic football. PeerJ..

[8] Jacobson I, Tegner Y (2007). Injuries among Swedish female elite football players: a prospective population study. Scand J Med Sci Sports.

[9] Bahr R, Krosshaug T (2005). Understanding injury mechanisms: a key component of preventing injuries in sport. Br J Sports Med.

[10] Verhagen EALM, Van Mechelen W (2010). Sport for all, injury prevention for all. Br J Sports Med.

[11] van Mechelen W, Hlobil H, Kemper HCG (1992). Incidence, severity, aetiology and prevention of sports injuries: a review of concepts. Sport Med An Int J Appl Med Sci Sport Exerc.

[12] Finch C (2006). A new framework for research leading to sports injury prevention. J Sci Med Sport.

[13] Fuller CW, Molloy MG, Bagate C, Bahr R, Brooks JHM, Donson H, Kemp SPT, McCrory P, McIntosh AS, Meeuwisse WH, Quarrie KL, Raftery M, Wiley P (2007). Consensus statement on injury definitions and data collection procedures for studies of injuries in rugby union. Clin J Sport Med.

[14] Fuller CW, Ekstrand J, Junge A, Andersen TE, Bahr R, Dvorak J, Hägglund M, McCrory P, Meeuwisse WH (2006). Consensus statement on injury definitions and data collection procedures in studies of football (soccer) injuries. Br J Sports Med.

[15] Orchard JW, Ranson C, Olivier B, Dhillon M, Gray J, Langley B, Mansingh A, Moore IS, Murphy I, Patricios J, Alwar T, Clark CJ, Harrop B, Khan HI, Kountouris A, Macphail M, Mount S, Mupotaringa A, Newman D, O’Reilly K, Peirce N, Saleem S, Shackel D, Stretch R, Finch CF (2016). International consensus statement on injury surveillance in cricket: a 2016 update. Br J Sports Med.

[16] Junge A, Engebretsen L, Alonso JM, Renström P, Mountjoy M, Aubry M, Dvorak J (2008). Injury surveillance in multi-sport events: the International Olympic Committee approach. Br J Sports Med.

[17] Mountjoy M, Junge A, Alonso JM, Clarsen B, Pluim BM, Shrier I, Van Den Hoogenband C, Marks S, Gerrard D, Heyns P, Kaneoka K, Dijkstra HP, Khan KM, Van Den Hoogenband C, Marks S, Gerrard D, Heyns P, Kaneoka K, Dijkstra HP, Khan KM (2016). Consensus statement on the methodology of injury and illness surveillance in FINA (aquatic sports). Br J Sports Med.

[18] Timpka T, Alonso JM, Jacobsson J, Junge A, Branco P, Clarsen B, Kowalski J, Mountjoy M, Nilsson S, Pluim B, Renström P, Rønsen O, Steffen K, Edouard P (2014). Injury and illness definitions and data collection procedures for use in epidemiological studies in Athletics (track and field): consensus statement. Br J Sports Med.

[19] Pluim BM, Fuller CW, Batt ME, Chase L, Hainline B, Miller S, Montalvan B, Renström P, Stroia KA, Weber K, Wood TO (2009). Consensus statement on epidemiological studies of medical conditions in tennis, april 2009. Clin J Sport Med.

[20] Turner M, Fuller CW, Egan D, Masson L, McGoldrick A, Spence A, Wind P, Gadot P-M (2012). European consensus on epidemiological studies of injuries in the thoroughbred horse racing industry. Br J Sports Med.

[21] Orchard JW, Newman D, Stretch R, Frost W, Mansingh A, Leipus A (2005). Methods for injury surveillance in international cricket. Br J Sports Med.

[22] Finch CF, Cook J (2014). Categorising sports injuries in epidemiological studies: the subsequent injury categorisation (SIC) model to address multiple, recurrent and exacerbation of injuries. Br J Sports Med.

[23] Theisen D, Frisch A, Malisoux L, Urhausen A, Croisier JL, Seil R (2013). Injury risk is different in team and individual youth sport. J Sci Med Sport.

[24] Brooks JHM, Fuller CW, Kemp SPT, Reddin DB (2005). Epidemiology of injuries in English professional rugby union: part 1 match injuries. Br J Sports Med.

[25] Ekstrand J, Hagglund M, Walden M (2011). Epidemiology of muscle injuries in professional football (soccer). Am J Sports Med.

[26] Toohey LA, Drew MK, Cook JL, Finch CF, Gaida JE (2017). Is subsequent lower limb injury associated with previous injury? A systematic review and meta-analysis. Br J Sports Med.

[27] Moore IS, Ranson C, Mathema P (2015). Injury risk in international rugby union: three-year injury surveillance of the Welsh national team. Orthapedic J Sport Med J Sport Med.

[28] Fortington LV, Worp H, Akker-scheek I, Finch CF (2017). Reporting multiple individual injuries in studies of team ball sports: a systematic review of current practice. Sports Med.

[29] Finch CF, Cook JL, Kunstler BE, Akram M, Orchard JW (2017). Subsequent injuries are more common than injury recurrences an analysis of 1 season of prospectively collected injuries in professional Australian football. Am J Sports Med.

[30] Knaap D, Stege J, Verhagen EA, Van W, Stubbe JH, Van Beijsterveldt AMMC, Van Der Knaap S, Stege J, Verhagen EA, Van Mechelen W, Backx FJG (2015). Injuries in professional male soccer players in the Netherlands: a prospective cohort study. J Athl Train.

[31] Toohey LA, Drew MK, Fortington LV, Finch CF, Cook JL (2018). An updated subsequent injury categorisation model (SIC-2.0): data-driven categorisation of subsequent injuries in sport. Sports Med.

[32] Hamilton GM, Meeuwisse WH, Emery CA, Shrier I (2011). Subsequent injury definition, classification, and consequence. Clin J Sport Med.

[33] Fuller CW, Bahr R, Dick RW, Meeuwisse WH (2007). A framework for recording recurrences, reinjuries, and exacerbations in injury surveillance. Clin J Sport Med.

[34] Moher D, Liberati A, Tetzlaff J, Altman DG, Altman D, Antes G, Atkins D, Barbour V, Barrowman N, Berlin JA, Clark J, Clarke M, Cook D, D’Amico R, Deeks JJ, Devereaux PJ, Dickersin K, Egger M, Ernst E, Gøtzsche PC, Grimshaw J, Guyatt G, Higgins J, Ioannidis JPA, Kleijnen J, Lang T, Magrini N, McNamee D, Moja L, Mulrow C, Napoli M, Oxman A, Pham B, Rennie D, Sampson M, Schulz KF, Shekelle PG, Tovey D, Tugwell P (2009). Preferred reporting items for systematic reviews and meta-analyses: the PRISMA statement (Chinese edition). J Chinese Integr Med.

[35] Meeuwisse WH, Tyreman H, Hagel B, Emery C (2007). A dynamic model of etiology in sport injury: the recursive nature of risk and causation. Clin J Sport Med.

[36] Downs SH, Black N (1998). The feasibility of creating a checklist for the assessment of the methodologi. J Epidemiol Community Health.

[37] Bjørneboe J, Bahr R, Andersen TE (2014). Gradual increase in the risk of match injury in Norwegian male professional football: a 6-year prospective study. Scand J Med Sci Sports.

[38] Dvorak J, Junge A, Derman W, Schwellnus M (2011). Injuries and illnesses of football players during the 2010 FIFA World Cup. Br J Sports Med.

[39] Eirale C, Hamilton B, Bisciotti G, Grantham J, Chalabi H (2012). Injury epidemiology in a national football team of the Middle East. Scand J Med Sci Sports.

[40] Eirale C, Tol JL, Smiley F, Farooq A, Chalabi H. Does ramadan affect the risk of injury in professional football? Clin J Sport Med. 2013;23:261–6 http://www.embase.com/search/results?subaction=viewrecord&from=export&id=L52509521;%5Cn10.1097/JSM.0b013e31828a2bfb;%5Cnhttp://sfx.ub.rug.nl:9003/sfx_local?sid=EMBASE&issn=1050642X&id=doi:10.1097/JSM.0b013e31828a2bfb&atitle=Does+ramadan+. 10.1097/JSM.0b013e31828a2bfb 23528844

[41] Ekstrand J, Van Dijk CN, Van Dijk CN (2013). Fifth metatarsal fractures among male professional footballers: a potential career-ending disease. Br J Sports Med.

[42] Ekstrand J, Waldén M, Hägglund M (2016). Hamstring injuries have increased by 4% annually in men’s professional football, since 2001: a 13-year longitudinal analysis of the UEFA Elite Club injury study. Br J Sports Med.

[43] Ekstrand J, Healy JC, Waldén M, Lee JC, English B, Hägglund M (2012). Hamstring muscle injuries in professional football: the correlation of MRI findings with return to play. Br J Sports Med.

[44] Ekstrand J, Hagglund M, Walden M. Injury incidence and injury patterns in professional football - the UEFA injury study. Br J Sports Med. 2009. 10.1136/bjsm.2009.060582 19553225

[45] Ekstrand J, Askling C, Magnusson H, Mithoefer K (2013). Return to play after thigh muscle injury in elite football players: implementation and validation of the Muich muscle injury classification. Br J Sports Med.

[46] Ekstrand J, Torstveit MK (2012). Stress fractures in elite male football players. Scand J Med Sci Sports.

[47] Ekstrand J, Lee JC, Healy JC (2016). MRI findings and return to play in football: a prospective analysis of 255 hamstring injuries in the UEFA Elite Club Injury Study. Br J Sports Med.

[48] Ergün M, Denerel HN, Nnet MSB, Ertat KA, Binnet MS, Ertat KA, Nnet MSB, Ertat KA (2013). Injuries in elite youth football players: a prospective three-year study. Acta Orthop Traumatol Turc.

[49] Gajhede-knudsen M, Ekstrand J, Magnusson H, Maffulli N (2013). Recurrence of Achilles tendon injuries in elite male football players is more common after early return to play: an 11-year follow-up of the UEFA Champions League injury study. Br J Sports Med.

[50] Gomez-Piqueras P, Gonzales-Villora S, Grassi A, Gojanovic B, Hagglund M, Walden M (2018). Are we making SMART decisions regarding return to training of injured football players? Preliminary results from a pilot study. Isokinet Exerc Sci.

[51] Hallen A, Ekstrand J (2014). Return to play following muscle injuries in professional footballers. J Sports Sci.

[52] Jones A, Jones G, Greig N, Bower P, Brown J, Hind K, Francis P (2019). Epidemiology of injury in English Professional Football players: a cohort study. Phys Ther Sport.

[53] Larruskain J, Lekue JA, Diaz N, Odriozola A, Gil SM (2018). A comparison of injuries in elite male and female football players: a five-season prospective study. Scand J Med Sci Sports.

[54] Lee JW-YJW, Mok K-MK, Chan HCHC-K, Yung PSPS-H, Chan KK-M (2014). A prospective epidemiological study of injury incidence and injury patterns in a Hong Kong male professional football league during the competitive season. Asia-Pacific J Sport Med Arthrosc Rehabil Technol.

[55] Lundblad M, Waldén M, Magnusson H, Karlsson J, Ekstrand J (2013). The UEFA injury study: 11-year data concerning 346 MCL injuries and time to return to play. Br J Sports Med.

[56] Nordström A, Nordström P, Ekstrand J (2014). Sports-related concussion increases the risk of subsequent injury by about 50% in elite male football players. Br J Sports Med.

[57] Noya Salces J, Gómez-Carmona PM, Gracia-Marco L, Moliner-Urdiales D, Sillero-Quintana M (2014). Epidemiology of injuries in First Division Spanish football. J Sports Sci.

[58] Petersen J, Thorborg K, Nielsen MB, Hölmich P, Ho P, Hospital A (2010). Acute hamstring injuries in Danish elite football: a 12-month prospective registration study among 374 players. Scand J Med Sci Sports.

[59] Ekstrand J, Krutsch W, Spreco A, Van Zoest W, Roberts C, Meyer T, Bengtsson H (2020). Time before return to play for the most common injuries in professional football: a 16-year follow-up of the UEFA Elite Club Injury Study. Br J Sports Med.

[60] Van Beijsterveldt AMCA, Stubbe JH, Schmikli SL, Van De Port IGL, Backx FJG, Anne-Marie van Beijsterveldt AMC, Stubbe JH, Schmikli SL, Van De Port IGL, Backx FJG (2015). Differences in injury risk and characteristics between Dutch amateur and professional soccer players. J Sci Med Sport.

[61] Hagglund M, Walden M, Ekstrand J, Waldén M, Ekstrand J (2012). Risk factors for lower extremity muscle injury in professional soccer: the UEFA injury study. Am J Sports Med.

[62] Hagglund M, Zwerver J, Ekstrand J (2011). Epidemiology of patellar tendinopathy in elite male soccer players. Am J Sports Med.

[63] Larruskain J, Celorrio D, Barrio I, Odriozola A, Gil SM, Fernandez-Lopez JR, Nozal R, Ortuzar I, Lekue JA, Aznar JM (2018). Genetic variants and hamstring injury in soccer: an association and validation study. Med Sci Sports Exerc.

[64] Bengtsson H, Ekstrand J, Waldén M, Hägglund M (2020). Few training sessions between return to play and first match appearance are associated with an increased propensity for injury: a prospective cohort study of male professional football players during 16 consecutive seasons. Br J Sports Med.

[65] Cross M, Kemp S, Smith A, Trewartha G, Stokes K (2016). Professional Rugby Union players have a 60% greater risk of time loss injury after concussion: a 2-season prospective study of clinical outcomes. Br J Sports Med.

[66] Fuller CW, Laborde F, Leather RJ, Molloy MG (2008). International Rugby Board Rugby World Cup 2007 injury surveillance study. Br J Sports Med.

[67] Fuller CW, Sheerin K, Targett S (2013). Rugby World Cup 2011: International Rugby Board injury surveillance study. Br J Sports Med.

[68] Kemp T, Brooks JHMM, Fuller CW, Kemp SPT, Hudson Z, Brooks JHMM, Fuller CW, Brooks DB (2008). J H M; Fuller, CW; Kemp, S P T; Redding, The epidemiology of head injuries in English professional rugby. Clin J Sport Med.

[69] Ma R, Lopez V, Weinstein MG, Chen JL, Black CM, Gupta AT, Harbst JD, Victoria C, Allen AA (2016). Injury profile of American women’s Rugby-7s. Med Sci Sports Exerc.

[70] Moore IS, Mount S, Mathema P, Ranson C (2018). Application of the subsequent injury categorisation model for longitudinal injury surveillance in elite rugby and cricket: intersport comparisons and inter-rater reliability of coding. Br J Sports Med.

[71] Pearce CJ, Brooks JHM, Kemp SPT, Calder JDF (2011). The epidemiology of foot injuries in professional rugby union players. Foot Ankle Surg.

[72] Rafferty J, Ranson C, Oatley G, Mostafa M, Mathema P, Crick T, Moore IS (2018). On average, a professional rugby union player is more likely than not to sustain a concussion after 25 matches. Br J Sports Med.

[73] Williams S, Trewartha G, Kemp S, Cross MJ, Brooks JHM, Fuller CW, Taylor AE, Stokes KA (2017). Subsequent injuries and early recurrent diagnoses in elite rugby union players. Int J Sports Med.

[74] Kenneally-Dabrowski C, Serpell BG, Spratford W, Lai AKM, Field B, Brown NAT, Thomson M, Perriman D (2019). A retrospective analysis of hamstring injuries in elite rugby athletes: more severe injuries are likely to occur at the distal myofascial junction. Phys Ther Sport.

[75] Toohey LA, Drew MK, Finch CF, Cook JL, Fortington LV (2019). A 2-year prospective study of injury epidemiology in elite Australian rugby sevens: exploration of incidence rates, severity, injury type, and subsequent injury in men and women. Am J Sports Med.

[76] Alonso JM, Junge A, Renstro P, Renström P, Engebretsen L, Mountjoy M, Dvorak J (2009). Sports injuries surveillance during the 2007 IAAF world athletics championships. Clin J Sport Med.

[77] Alonso JM, Tscholl PM, Engebretsen L, Mountjoy M, Dvorak J, Junge A (2010). Occurrence of injuries and illnesses during the 2009 IAAF World Athletics Championships. Br J Sports Med.

[78] Alonso JM, Edouard P, Fischetto G, Adams B, Depiesse F, Mountjoy M (2012). Determination of future prevention strategies in elite track and field: analysis of Daegu 2011 IAAF Championships injuries and illnesses surveillance. Br J Sports Med.

[79] Edouard P, Depiesse F, Hertert P, Branco P, Alonso JM (2013). Injuries and illnesses during the 2011 Paris European Athletics Indoor Championships. Scand J Med Sci Sports.

[80] Edouard P, Depiesse F, Branco P, Alonso J-M (2014). Analyses of Helsinki 2012 European athletics championships injury and illness surveillance to discuss elite athletes risk factors. Clin J Sport Med.

[81] Junge A, Engebretsen L, Mountjoy M, Alonso JM, Renström PAFH, Aubry MJ, Dvorak J (2009). Sports injuries during the Summer Olympic Games 2008. Am J Sports Med.

[82] Ekstrand J, Hagglund M, Walden M, Ekstrand J (2006). Previous injury as risk factor for injury in elite football: a prospective study over two consecutive seasons. Br J Sports Med.

[83] A. Arnason, S. Sigurdsson, A. Gudmundsson, I. Holme, L. Engebretsen, R. Bahr, Risk factors for injuries in football, Am. J. Sports Med. 32 (2004). 10.1177/0363546503258912 14754854

[84] J.W. Orchard, Intrinsic and extrinsic risk factors for muscle strains in Australian football, Am. J. Sports Med. 29 (2001). 10.1177/03635465010290030801 11394599

